# Short–Term Preconditioning With Blood Flow Restricted Exercise Preserves Quadriceps Muscle Endurance in Patients After Anterior Cruciate Ligament Reconstruction

**DOI:** 10.3389/fphys.2018.01150

**Published:** 2018-08-24

**Authors:** Tina Žargi, Matej Drobnič, Klemen Stražar, Alan Kacin

**Affiliations:** ^1^Department of Physiotherapy, Faculty of Health Sciences, University of Ljubljana, Ljubljana, Slovenia; ^2^Department of Orthopedic Surgery, University Medical Centre Ljubljana, Ljubljana, Slovenia

**Keywords:** disuse muscle atrophy, blood flow restricted exercise, arthroscopic ACL reconstruction, ischemic preconditioning, quadriceps femoris, muscle endurance, muscle perfusion

## Abstract

Surgical ACL reconstruction performed with a tourniquet induces compression and ischemic stress of the quadriceps femoris (QF) muscle which can accelerate postoperative weakness. Given that low-load blood flow restricted (BFR) exercise is potent in enhancing muscle oxygenation and vascular function, we hypothesized that short-term preconditioning with low-load BFR exercise can attenuate QF muscle endurance deterioration in the postoperative period. Twenty subjects undergoing arthroscopic ACL reconstruction performed 5 exercise sessions in the last 8 days prior to surgery. They were assigned into either BFR group, performing low-load BFR knee-extension exercise, or SHAM-BFR group, replicating equal training volume with sham occlusion. Blood flow (near-infrared spectroscopy) and surface EMG of QF muscle during sustained isometric contraction at 30% of maximal voluntary isometric contraction (MVIC) torque performed to volitional failure were measured prior to the intervention and again 4 and 12 weeks after surgery. There was an overall decrease (*p* = 0.033) in MVIC torque over time, however, no significant time-group interaction was found. The time of sustained QF contraction shortened (*p* = 0.002) in SHAM-BFR group by 97 ± 85 s at week 4 and returned to preoperative values at week 12. No change in the time of sustained contraction was detected in BFR group at any time point after surgery. RMS EMG amplitude increased (*p* = 0.009) by 54 ± 58% at week 4 after surgery in BFR group only. BFm increased (*p* = 0.004) by 52 ± 47% in BFR group, and decreased (*p* = 0.023) by 32 ± 19% in SHAM-BFR group at week 4 after surgery. Multivariate regression models of postoperative changes in time of sustained QF contraction revealed its high correlation (*R*^2^ = 0.838; *p* < 0.001) with changes in BFm and RMS EMG in the SHAM-BFR group, whereas no such association was found in the BFR group. In conclusion, enhanced endurance of QF muscle was triggered by combination of augmented muscle fiber recruitment and enhanced muscle perfusion. The latter alludes to a preserving effect of preconditioning with BFR exercise on density and function of QF muscle microcirculation within the first 4 weeks after ACL reconstruction.

## Introduction

Quadriceps femoris (QF) muscle weakness is the major cause of poor functional status of patients following otherwise successful anterior cruciate ligament (ACL) reconstruction (Lindstrom et al., 2013; Thomas et al., 2015). Hence, the primary goal of postoperative rehabilitation is to restore normal muscle activation and function as soon as possible. However, despite best efforts from both patient and physiotherapist during the rehabilitation process, considerable impairments of QF muscle often persist for several months after the reconstruction (Kim et al., 2010). The deficits in muscle strength and endurance contribute to altered movement patterns of the involved limb and thus increase the risk of early onset of knee osteoarthritis (Palmieri-Smith and Thomas, 2009). Rather low efficiency of standard postoperative rehabilitation has diverted the focus of clinical research in recent years toward the so-called preconditioning, or prehabilitation, exercise programs. They aim to increase lower limb muscle strength prior to surgery, which is believed to substantially attenuate the deterioration of muscle function in the aftermath. Although temporary enhancements of muscle strength and function can be clearly gained with various preconditioning programs (Keays et al., 2006; Hartigan et al., 2009), there is little evidence to support their alleged protective effect against muscle deconditioning in the postoperative period (Shaarani et al., 2013; Kim et al., 2015). The impetus for utilizing preconditioning programs comes from several studies showing strong positive associations between preoperative strength levels of knee muscles, QF in particular, and the successful long-term outcome of ACL reconstruction (Eitzen et al., 2009; Logerstedt et al., 2013). However, focusing only on maximal muscle strength gains prior to surgery may not give the best results. Our recent analysis has shown that the level of preoperative QF muscle endurance, and not its maximal strength, is the strongest predictor of muscle atrophy in the first 4 weeks after ACL reconstruction (Grapar Žargi et al., 2017). Enhancing preoperative QF muscle endurance may therefore be more important for effective protection against postoperative deconditioning in these patients.

Why some patients develop severe postoperative QF muscle weakness is not fully understood. Development of postoperative muscle atrophy and dysfunction is a multifactorial phenomenon, which is mainly caused by reduced limb loading and arthrogenic muscle inhibition driven by pain and joint swelling (Rice et al., 2014, 2015). In addition, a latent ischemic-reperfusion injury, triggered by prolonged arterial occlusion during surgery, augments QF atrophy in the early postoperative period (Appell et al., 1993; Daniel et al., 1995). It can be estimated from animal models that irreversible damage to skeletal muscle develops only after 3–6 h of complete ischemia (Blebea et al., 1987; Belkin et al., 1988). However, smaller-scale edema of the vastus lateralis muscle cells and surrounding microcirculation is initiated after only 15 min of surgery performed with a tourniquet, and progresses to severe damage and death of some myocytes after 90 min of ischemia (Appell et al., 1993). Utilizing a standard ischemic preconditioning (IPC) protocol, comprised of several 3–5 min intervals of complete blood flow restriction intermittent by equal periods of reperfusion, on a resting skeletal muscle before its exposure to prolonged ischemia can reduce gross muscle damage and increase cell survival (Sullivan et al., 2009; Murphy et al., 2010). The IPC has proven successful in attenuating muscle damage and atrophy following extremity surgery in animals (Gürke et al., 1996; Papanastasiou et al., 1999); however, no evidence of its effectiveness in human surgery is currently available in the literature. Interestingly, a frequent application (twice daily) of IPC was reported to prevent atrophy and weakness of several lower limb muscles during 14-day experimental ankle cast immobilization in otherwise healthy young males (Kubota et al., 2008).

Another muscle conditioning method, utilizing intermittent muscle blood flow restriction similar to standard IPC protocol, but in combination with low-load resistance exercise (low-load BFR exercise), has been extensively studied in recent decades. In healthy humans, the low-load BFR exercise was proven to be as effective as conventional high-load resistance exercise in increasing muscle mass and strength (Takarada et al., 2004; Abe et al., 2006). It was also demonstrated to be superior in enhancing local muscle endurance to equal exercise with normal blood flow (Takarada et al., 2002; Kacin and Strazar, 2011; Sousa et al., 2017). The endurance boosting effect of BFR exercise is closely related to enhanced muscle vascular function (Patterson and Ferguson, 2010; Hunt et al., 2013) and oxygenation (Kacin and Strazar, 2011). It was also shown to attenuate muscle atrophy during experimental limb unloading (Clark et al., 2006) and in patients after ACL reconstruction (Ohta et al., 2003), although evidence of no effect for the latter also exist (Iversen et al., 2016). Despite shorter time under occlusion compared to IPC, and the fact that complete arterial occlusion is usually not achieved prior to commencement of muscle contractions, low-load BFR exercise can nevertheless pose a short, but very intensive ischemic stimulus to the muscle tissue. Depletion of oxygen and accumulation of metabolites in the muscle are augmented by vigorous muscle activity (Suga et al., 2010), while muscle perfusion is progressively decreased due to build-up of intra-muscular pressure (Kacin et al., 2015), which can result in complete arterial occlusion in the muscle toward the end of exercise set. The intensity of ischemia-reperfusion stimulus can be modulated by various parameters of low-load BFR exercise protocol. If performed with combination of wider cuffs and higher pressure, longer time under occlusion and reperfusion periods between sets, BFR exercise induces stimulus similar to standard IPC, while, at the same time, provides substantial neuromuscular and metabolic stimuli of resistance exercise. We therefore presume that simultaneous activation of these two distinct, but partly overlapping, physiological stimuli would most effectively prevent postoperative dysfunction of QF muscle. However, our first study of short-term preconditioning of QF muscle with such low-load BFR exercise protocol has not detected a significant attenuation of atrophy, or loss of maximal strength, in the first 12 weeks following ACL reconstruction (Grapar Zargi et al., 2016). When analyzing possible reasons for no effect of the intervention, we realized that by focusing on maximal muscle strength we may have overlooked its more plausible preventative effect. Given that low-load BFR exercise is very potent in enhancing skeletal muscle endurance and vascular function in healthy subjects, it is likely that the preconditioning with BFR exercise would have more protective effect on the same in patients exposed to prolonged surgery with a tourniquet.

We thus hypothesized in the present study that the primary effect of preconditioning with low-load BFR exercise would be to preserve QF muscle endurance and microvascular function during the first 12 weeks after ACL reconstruction and that the changes in endurance are strongly associated with changes in muscle blood flow and pattern of muscle activation.

## Materials and methods

### Ethical approval

The study protocol was approved by the Republic of Slovenia National Medical Ethics Committee (permit No. 62/05/12, date of approval: 05/30/2012) and carried out in accordance with the Declaration of Helsinki. All patients included signed a written consent of voluntary participation after receiving detailed written and oral information on the study. Patient recruitment and surgical procedures took place at Orthopedic Department of University Medical Centre Ljubljana, whereas all performance tests and exercise training interventions were conducted at the university-based Laboratory of Physiotherapy Research.

### Study population

This study is a prospective, single-center, quasi-randomized trial controlled by SHAM-BFR intervention. Volunteers were selected and recruited among patients scheduled for an arthroscopic ACL reconstruction with ipsilateral hamstrings autographs ACL reconstruction. In total, 83 patients were assessed for eligibility to enter the study. Initially 24 patients were recruited for the study and 20 patients (16 males and 4 females) fully completed the protocol. Four patients were excluded from the final analysis due to additional surgical procedures requiring modification of post-operative rehabilitation program. The inclusion criteria were: age from 18 to 45 years, ACL tear of more than 6 months with sufficient range of motion to perform exercise (active extension deficit < 5°, active flexion ≥120°), pain level during exercise ≤ 20 on Visual Analogue Scale (0–100 mm) and no previous surgeries to the affected knee. The exclusion criteria were any concomitant intra-articular pathology that would require open surgery or prolonged postoperative unloading, a significant damage to the articular cartilage, additional neuromuscular impairments, spine or other lower limb injuries, presence or history of any vascular diseases or deep vein thrombosis.

### Surgical intervention and rehabilitation

All patients underwent surgery at the same institution under spinal anesthesia by two experienced orthopedic surgeons. During surgery, a pneumatic tourniquet inflated to minimum of 300 mmHg was used on the proximal part of the thigh to completely occlude the blood flow. Surgery was performed on five dominant and five non-dominant legs in each group, where the limb dominance was determined according to the patients' self-reported preference for kicking and take-off activities. An arthroscopic single bundle ACL reconstruction was performed using a double stranded ipsilateral semitendinosus-gracilis autograft. In 12 out of 20 patients, additional partial meniscectomy was performed during surgery. Postoperative rehabilitation began on the first postoperative day, when the patients were allowed to ambulate with full weight-bearing and received functional neuromuscular electrical stimulation of thigh muscles combined with volitional isometric contractions. No postoperative braces or crutches were used. During the first 5 weeks, all patients followed identical postoperative protocol (3 times per week, cryotherapy, passive and active ROM exercises, manual joint mobilization, open and closed kinetic chain resistance exercises, balance and proprioceptive exercises) supervised by appointed physiotherapist at the designated outpatient rehabilitation unit, followed by a 14-day intensified rehabilitation program (daily sessions of individual and group therapeutic exercise for gaining full knee ROM, muscle strength and joint stability) in a spa resort. The appointed physiotherapists in both facilities were blinded of the patients' group.

### Intervention

The short-term preconditioning intervention with low-load BFR exercise was designed to (i) deliver an IPC-induced protective effect on subsequent ischaemic-induced injury and (ii) increase muscle strength and endurance, as reviewed in the introduction. To induce ischemia-reperfusion stimulus similar to standard IPC, the BFR exercise protocol utilized in our patients comprised a combination of wider cuffs and moderate pressure, longer time under occlusion and longer reperfusion periods between sets. To emphasize the preventative effects of preconditioning, the last training session was performed within 48 h prior to surgery. To overcome patients' low tolerance for mechanical joint loading and assure the minimal required exercise volume and intensity of each session (Wernbom et al., 2009), multiple sets of exercise with very low resistance and high number of repetitions performed to volitional failure was used. Patients were asked not to change their routine regarding regular daily physical activities during the intervention period. The same BFR exercise protocol is described in detail in previous study from our lab (Grapar Zargi et al., 2016).

Patients conducted 5 exercise sessions, equally distributed during the last 8 days before the surgery. One patient group performed blood flow restricted exercise (BFR group) and the other group performed the exercise with sham blood flow restriction (SHAM-BFR group). A counterbalanced quasi-randomization of the patients was performed to match patients between groups by sex, overall score of Lysholm Knee Scoring Scale for patient's self-assessment of knee function (Tegner and Lysholm, 1985) and body mass index; thus, the first 2 patients were assigned to BFR group and the next two to SHAM-BFR group. This proceeding was repeated until each group comprised 10 patients who completed the entire experimental protocol. This protocol ensured equal volume of mechanical work performed by the two groups.

The patients performed unilateral resisted knee extension in an open kinetic chain on a leg-extension machine (Sokol Gym, Slovenia, EU) with ACL deficient leg only. The non-injured leg was not trained and served as a control for calculation of strength deficits. Patients in BFR group performed 6 sets of knee-extension exercise to volitional failure at each exercise session. A metronome was set to 56 bpm to determine the rhythm and speed; one beat for eccentric and one beat for concentric phase of muscle contraction. The 40-repetition maximum was set individually for each patient before the first exercise session by trial and error. Only after the initial warm-up with 10–15 repetitions with 0.5 kg load, were the tourniquets inflated to 150 mmHg and after 30 s of resting occlusion the patient started to exercise. After the first, the third and the fifth set, 45 s of rest without reperfusion was allowed. Only after the second and the fourth set, the tourniquets were deflated to allow 90 s of reperfusion. The patients in SHAM-BFR group followed the same exercise protocol and replicated the number of repetitions per set achieved by his/her pair from the BFR group.

Muscle blood flow was restricted with a 14-cm wide contoured pneumatic tourniquet cuff (VariFit Conture Thigh Cuff, Delfi Medical Innovations, Canada) placed around the proximal part of the thigh and connected to a pressure regulating system (Portable Tourniquet System, Delfi Medical Innovations, Canada). A uniform cuff pressure of 150 mmHg used in the BFR group was set based on results of series of prior pilot experiments performed in our lab on healthy subjects. This pressure level allows for maximal QF muscle deoxygenation at the end of each exercise set (measured with NIRS), without compromising normal muscle activation due to pain and discomfort elicited at the site of tissue compression (authors' unpublished data). In SHAM-BFR group, the occlusion pressure was set to only 20 mmHg, therefore not restricting muscle blood flow.

### Outcome measures

Assessment of muscle strength and endurance comprised measurements of maximal volitional isometric contraction (MVIC) torque and time of sustained submaximal isometric contraction, surface EMG of vastus medialis muscle and oxygenation and hemoglobin kinetics of vastus lateralis muscle. The measurements were performed prior to the preconditioning protocol (PRE) and repeated at week 4 (POST wk4), with exclusion of MVIC torque, and at week 12 (POST wk12) after surgery.

#### Muscle maximal isometric torque

The patients performed MVIC torque measurement on static dynamometer (Isometric Knee Dynamometer, S2P d.o.o., Ljubljana, Slovenia) in a sitting position with 60° of knee flexion and stabilized over pelvis with security belt. Loading of ACL has been shown to be minimal in this joint position (Escamilla et al., 2012), which allowed safe testing also postoperatively. Three attempts of 3–4 s MVIC were performed with 3 min rest period in-between to avoid muscle fatigue. Strong verbal encouragement was systematically given to motivate subjects. The highest torque of the three attempts averaged over 1 s was used for further analyses.

#### Muscle isometric endurance

The QF endurance test was performed in the same position as described above. The 30% of preoperative MVIC torque was calculated for each individual and set as a target for evaluating endurance. Based on our pilot experiments, this level of sustained contraction proved to be demanding enough and feasible in both preoperative and postoperative periods, which allowed comparison of the contraction times at equal absolute load at all three time points. A line marking the target of 30% of individual's MVIC torque was provided on a computer screen set in front of the patient for visual feedback. The patient followed the target line as closely as possible during the test. Another line marking the lowest limit for test termination at 90% of the target value was also shown on the screen. The test was terminated at patient's volitional failure, or if muscle force dropped below the limit for more than 3 s despite strong verbal encouragement. The time of sustained contraction at target was used as measure of the endurance.

#### Surface electromyography

The activation of vastus medialis muscle was measured by surface EMG during the endurance test. Electrodes were positioned according to SENIAM standards (Hermens et al., 2000) to avoid overlap of the innervation zones and cross-talk of the muscles. Skin was shaved, lightly abraded and cleaned with alcohol to reduce electrical impedance < 5,000 Ohms. A pair of bipolar surface electrodes Ag-AgCl (Red Dot 2560, 3M, USA) was placed longitudinally on the muscle belly. EMG activity was amplified with four-channel tethered remote monitoring unit at a sampling rate of 1,000 Hz, input impedance 2 MΩ, bandwidth of 1–500 Hz (TEL 100C and MP150WS, Biopac Systems, USA). The raw EMG data were filtered with a high pass infinite impulse response digital filter at a cutoff frequency of 20 Hz (Acqknowledge 3.9.2., Biopac Systems Inc.). Root mean square (RMS) smoothening of the filtered signal with 3,000 ms time window was used to quantify EMG amplitude. The frequency and power analysis included fast Fourier transformation of the filtered EMG signal, followed by median frequency calculation (F_med_) for the entire contraction time. Standard normalization of EMG amplitude to MVIC values was neither feasible nor appropriate due to potential injury of reconstructed ACL graft at POST WK4 during maximal QF contraction and substantial decrease in postoperative MVIC torque. EMG amplitude at the same absolute torque, corresponding to 30% of the initial MVIC torque, was compared at all three time points.

#### Muscle blood flow

Muscle blood flow (BF_m_) in vastus lateralis muscle was measured with near-infrared spectroscopy (NIRS) during the endurance test (Oxymon Mk III, Artinis Medical Systems, Arnheim, The Netherlands), based on absorption of infrared light in the muscle tissue (Ferrari et al., 2004). Concentrations of oxygenated ([O_2_Hb]) hemoglobin and deoxygenated hemoglobin ([HHb]) in the muscle were measured with 760 and 850 nm wavelengths, respectively. The skin on the muscle belly was shaved, lightly abraded and cleaned with alcohol before plastic holder for two optodes was fixed to the skin with double-sided adhesive tape. The interoptode distance was set between 35 and 50 mm, depending on the subject's skinfold thickness at the site of optode placement. To minimize the influence of daily variations, the values are reported as deltas (Δ). Resting values were acquired during 10 min of quite rest in the same sitting position as used for testing. To assess muscle blood flow a 30-s venous occlusion (cuff pressure = 60 mmHg) was repeated twice with 15 s pause in-between. The data were averaged over the two measures. To obtain the resting values the procedure was performed from 4 to 6 min of the rest period. To obtain post-exercise values the procedure was performed immediately after the endurance test, starting within 5 s after cessation of sustained isometric contraction. Signals were acquired at a sampling rate of 10 Hz and recorded. The raw signal was filtered with 2-sec moving average (Oxysoft 2.0.47 software, Artinis Medical Systems, Arnheim, The Netherlands) to remove movement artifacts. Blood flow was calculated using the following formula (Van Beekvelt et al., 2001):

$${\text{BF}}_{\text{m}}=(((\Delta [\text{tHb}]\cdot 60)/(([\text{Hb}]\cdot 1000/4))\cdot 1000)/10$$

where [tHb] is expressed in μM·s^−1^ and converted to milliliters of blood per minute per 100 milliliters of tissue (mL·min^−1^·100mL^−1^) and group reference values for hemoglobin concentration ([Hb] in mmol·L^−1^) of males and females obtained from the literature are used. The molecular weight of Hb (64.458 g·mol^−1^) and the molecular ratio between Hb and O_2_ (1:4) was also taken into account. The intra-observer coefficient of variation was 8.2%.

### Statistical analysis

All statistical analyses were performed with Statistica software (Version 12, StatSoft Inc., Tulsa, Oklahoma, USA). A threshold of significance was set at α < 0.05 for all tests. Unless stated otherwise, variables are expressed as means ± standard deviation (SD).

#### Sample size estimation

Estimation of minimal sample size at statistical power level of ≥0.80 (β ≤ 20%) was calculated for two-way ANOVA test based on standardized effective size for the main observed outcome measure of QF muscle endurance, i.e., the time of sustained isometric contraction. A predicted preoperative mean of 190 s with standard deviation of 30 s, determined by our pilot experiments, was used for calculations. By assuming an interaction of 30% difference between the groups over time, the estimated minimal number of subjects was nine.

#### Comparison of means

Normality of distribution of each data set was analyzed with Shapiro-Wilk test and parametric statistical analysis was deemed appropriate. The initial patients' characteristics of the groups were tested with the Student's *t*-test. Comparison of means in contraction time, MVIC torque, RMS EMG, F_med_ and BF_m_ was made with two-way (group × time) ANOVA with repeated measures on the time factor. In case of significant main effect, the *post hoc* pairwise comparisons were made with the Tukey's honestly significant difference test.

#### Regression analyses

The regression analysis aimed to elucidate the extent of association between changes in QF endurance and changes in muscle activation and perfusion. A multivariable linear regression analysis of changes (Δ) in time of sustained contraction as the dependent variable was performed first from the pooled data of both groups and then separately for each group. The Δ RMS EMG and Δ BF_m_ were calculated for periods PRE to POST WK4 and POST WK4 to POST WK12. Both parameters were simultaneously introduced to the models as independent variables. The coefficient of determination (*R*^2^), model's intercept, regression coefficient (b), standardized regression coefficient (β) and *p*-value were computed and reported. To assess independent association between dependent and each independent variable, the univariate linear regressions were performed and Pearson's correlation coefficients (r) calculated.

## Results

There were no significant differences in body mass index (BFR = 24.3 ± 3.9 kg · m^−2^; SHAM-BFR = 23.9 ± 2.9 kg · m^−2^), age (BFR = 34 ± 6 years; SHAM-BFR = 35 ± 5 years) and Lysholm composite score (BFR = 78 ± 11; SHAM-BFR = 76 ± 16) between the groups. The average duration of the surgery was 71 min (range 52–113 min) with no significant difference between the groups (BFR = 69 ± 9 min; SHAM-BFR = 71 ± 14 min).

### Muscle isometric strength and endurance

**Figure 1:**
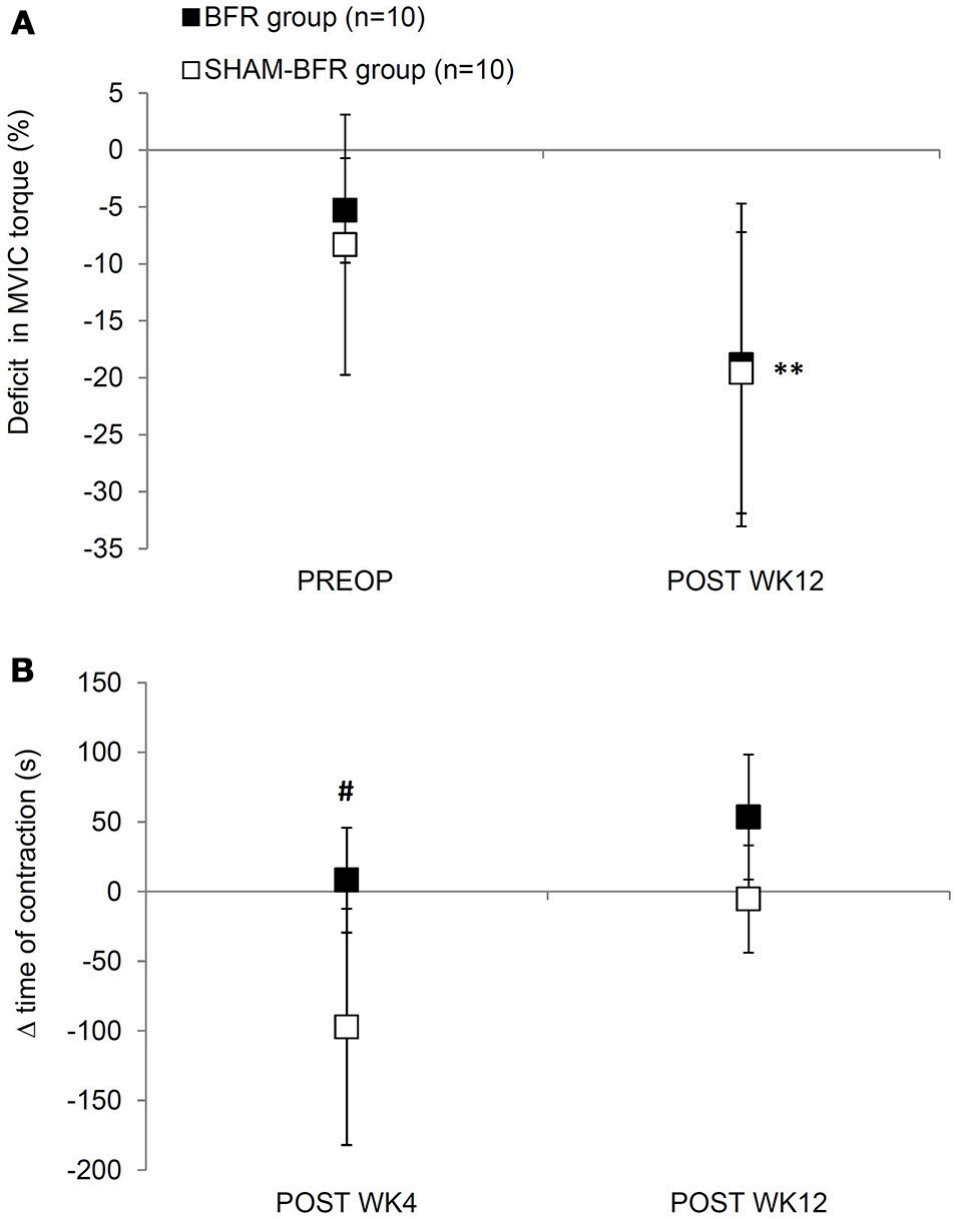
Mean (SD) deficits in MVIC torque **(A)** and changes (Δ) in time of sustained contraction at 30% MVIC torque **(B)** of quadriceps femoris muscle of the affected leg in BFR and SHAM-BFR group prior to (PREOP), at 4 weeks (POST WK4) and at 12 weeks (POST WK12) after surgery. ^#^denotes statistical difference between groups at *p* < 0.05. **denotes statistical difference compared to PREOP values at *p* < 0.01.

Absolute values of QF strength and endurance outcome measures and results of statistical analysis are presented in Table 1. There was an overall decrease in absolute MVIC torque over time, however no significant time-group interaction was found. Likewise, there was an overall change in MVIC torque deficit of OP leg in time, with non-significant time-group interaction. Twelve weeks after surgery, the deficit increased to −19 ± 14% in BFR group and −20 ± 12% in SHAM-BFR group, with no difference between the groups (Figure 1A).

**Table 1:** Mean (SD) values of quadriceps femoris muscle strength, endurance, EMG activity and blood flow in BFR and SHAM-BFR group during the 12-week postoperative period.

	**BFR group (*****n*** = **10)**			**SHAM-BFR group (*****n*** = **10)**			***p*****-value**		
	**PREOP**	**POST WK4**	**POST WK12**	**PREOP**	**POST WK4**	**POST WK12**	**Time**	**Group**	**Interaction**
**MUSCLE STRENGTH AND ENDURANCE**									
MVIC torque (Nm)	238 ± 83	/	196 ± 48	210 ± 44	/	181 ± 34	0.002	0.350	0.519
Time of contraction (s)	145 ± 47	153 ± 54	199 ± 71	192 ± 93	95 ± 90	187 ± 94	< 0.001	0.902	0.014
**EMG ACTIVITY AND MUSCLE PERFUSION**									
RMS EMG (mV)	0.274 ± 0.163	0.391 ± 0.293	0.256 ± 0.160	0.224 ± 0.096	0.187 ± 0.103	0.210 ± 0.086	0.049	0.125	0.001
F_med_ (s)	53.2 ± 10.9	45.7 ± 10.1	45.9 ± 4.4	49.9 ± 5.9	41.7 ± 5.8	44.1 ± 3.7	< 0.001	0.286	0.730
BF_m_ (mL. min^−1^. 100mL^−1^)	1.26 ± 0.34	1.92 ± 0.81	1.49 ± 0.75	1.53 ± 0.44	0.97 ± 0.20	1.36 ± 0.32	0.907	0.176	< 0.001

*MVIC, maximal volitional isometric contraction; RMS EMG, root mean square of electromyograhic amplitude; F_med_, median frequency of EMG spectrum; BF_m_, muscle blood flow; POST WK4, 4 weeks after surgery; POST WK12, 12 weeks after surgery; BFR, blood flow restriction; SHAM-BFR, sham blood flow restriction*.

The time of sustained contraction showed significant interaction of time and group factors (Table 1). It was significantly shorter (*p* < 0.001) in SHAM-BFR group at POST WK4 compared to the baseline value, whereas in BFR group it did not decrease significantly, resulting in a significantly different (*p* = 0.029) change in the parameter between the groups at POST WK4. At POST WK12, the time of sustained contraction remained at similar level in BFR group and returned to preoperative values in SHAM-BFR group (Figure 1B).

### Surface EMG and muscle blood flow

**Figure 2:**
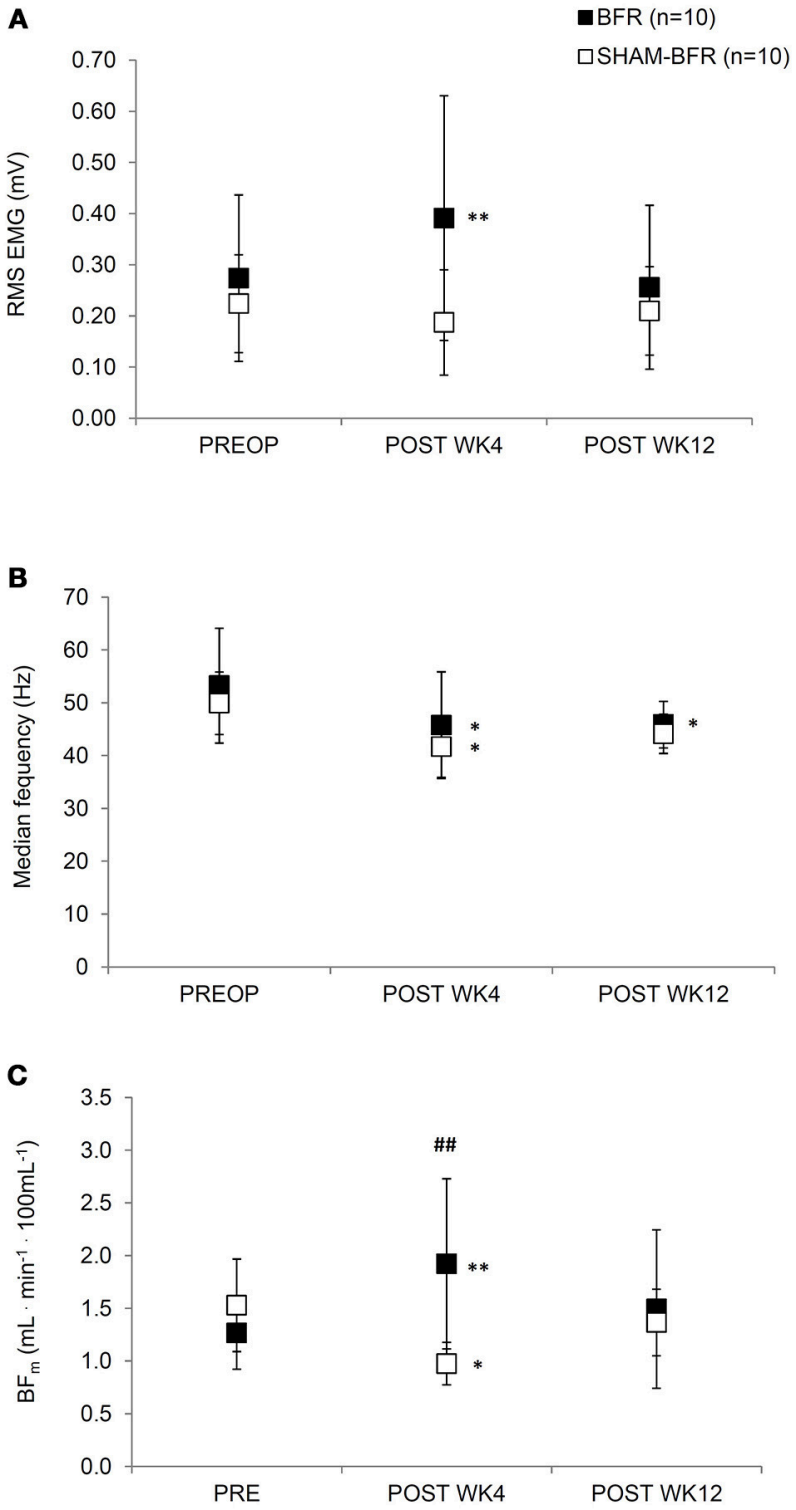
Mean (SD) values of root mean square EMG amplitude **(A)**, median EMG frequency **(B)** and blood flow **(C)** of the affected leg during sustained contraction of quadriceps femoris muscle at 30% MVIC torque for both BFR and SHAM-BFR group prior to (PREOP), at 4 weeks (POST WK4) and at 12 weeks (POST WK12) after surgery. ^##^denotes statistical difference between groups at *p* < 0.01. *,**denote statistical difference compared to PREOP values at *p* < 0.05 and *p* < 0.01, respectively.

Absolute values and results of statistical analysis of surface EMG and muscle blood flow are presented in Table 1. The amplitude of RMS EMG showed significant (*p* = 0.001) interaction of time and group factors. It significantly (*p* = 0.009) increased in BFR group by 54 ± 58% at POST WK4 and returned to preoperative values at POST WK12. No significant difference in RMS EMG was noted in SHAM-BFR at any postoperative time point. There was a tendency (*p* = 0.057) for significant difference in the amplitude of RMS EMG between groups at POST WK4 (Figure 2A).

The F_med_ showed no significant (*p* = 0.730) interaction of time and group factors. F_med_ decreased by 14 ± 6% in BFR group (*p* = 0.013) and by 16 ± 12% in SHAM-BFR group (*p* = 0.005) at POST WK4 and tended to remain lowered at POST WK12 (BFR = 12 ± 13 %, *p* = 0.016; SHAM-BFR = 11 ± 10%, *p* = 0.086). There were no significant differences between the groups at any timepoint (Figure 2B).

The BF_m_ showed significant (*p* < 0.001) interaction of time and group factors. BF_m_ significantly increased by 52 ± 47% in BFR group (*p* = 0.004), and decreased by 32 ± 19% in SHAM-BFR group (*p* = 0.023), at POST WK4. There was a significant difference (*p* = 0.004) in the BF_m_ between groups at POST WK4 (Figure 2C).

### Models of postoperative change in muscle endurance

A multiple regression model of pooled group data of Δ time of contraction of QF in the postoperative period revealed a significant (*p* = 0.006) relationship (*R*^2^ = 0.245) with the following Equation [1]:

(1) $$\begin{array}{l} \Delta \text{Time of contraction }=(-25.453\cdot \Delta \;\text{RMS EMG})+(65.500\cdot \Delta \\ \text{ BFm})+10.718 \end{array}$$

As shown in Table 2, the Δ RMS EMG had small and non-significant (*p* = 0.845) negative weight, while Δ BF_m_ had large and significant (*p* = 0.014) positive weight. The multiple regression model of Δ time of contraction of QF for the BFR group revealed a non-significant (*p* = 0.826) and weak relationship (*R*^2^ = 0.002) between the variables (Table 2). In contrast, the multiple regression model of Δ time of contraction of QF for the SHAM-BFR group revealed a significant (*p* < 0.001) and strong relationship (*R*^2^ = 0.838) with the following Equation [2]:

(2) $$\begin{array}{l} \Delta \text{Time of contraction}=(448.536\cdot \Delta \text{ RMS EMG})+(130.149\cdot \Delta \\ \text{ BFm})+10.994 \end{array}$$

As shown in Table 2, the both Δ RMS EMG (*p* = 0.002) and Δ BF_m_ (*p* < 0.001) had large and significant positive weight.

**Table 2:** Multivariate linear regression models of change in quadriceps femoris (QF) muscle time of submaximal isometric contraction after ACL reconstruction for pooled group data and separately for BFR and SHAM-BFR group.

	**β ± SE**	**b ± SE**	***p*-value**
**POOLED GROUP DATA**			
Intercept		10.718 ± 13.012	0.415
Δ RMS EMG (mV)	−0.040 ± 0.202	−25.453 ± 129.510	0.845
Δ BF_m_ (mL. min^−1^. 100 mL^−1^)	0.522 ± 0.202	65.501 ± 25.378	0.014
**BFR GROUP (*****n*** = **10)**			
Intercept		25.065 ± 9.534	0.018
Δ RMS EMG (mV)	−0.276 ± 0.449	−61.409 ± 99.882	0.547
Δ BF_m_ (mL. min^−1^. 100 mL^−1^)	0.210 ± 0.449	9.791 ± 20.939	0.646
**SHAM-BFR GROUP (*****n*** = **10)**			
Intercept		10.994 ± 11.882	0.368
Δ RMS EMG (mV)	0.400 ± 0.110	448.536 ± 122.931	0.002
Δ BF_m_ (mL. min^−1^. 100 mL^−1^)	0.661 ± 0.110	130.149 ± 21.568	< 0.001

*Δ RMS EMG, change in root mean square EMG amplitude; Δ BF_m_, change in muscle blood flow; BFR, blood flow restriction; SHAM-BFR, sham blood flow restriction; SE, standard error*.

### Univariate regression analysis

**Figure 3:**
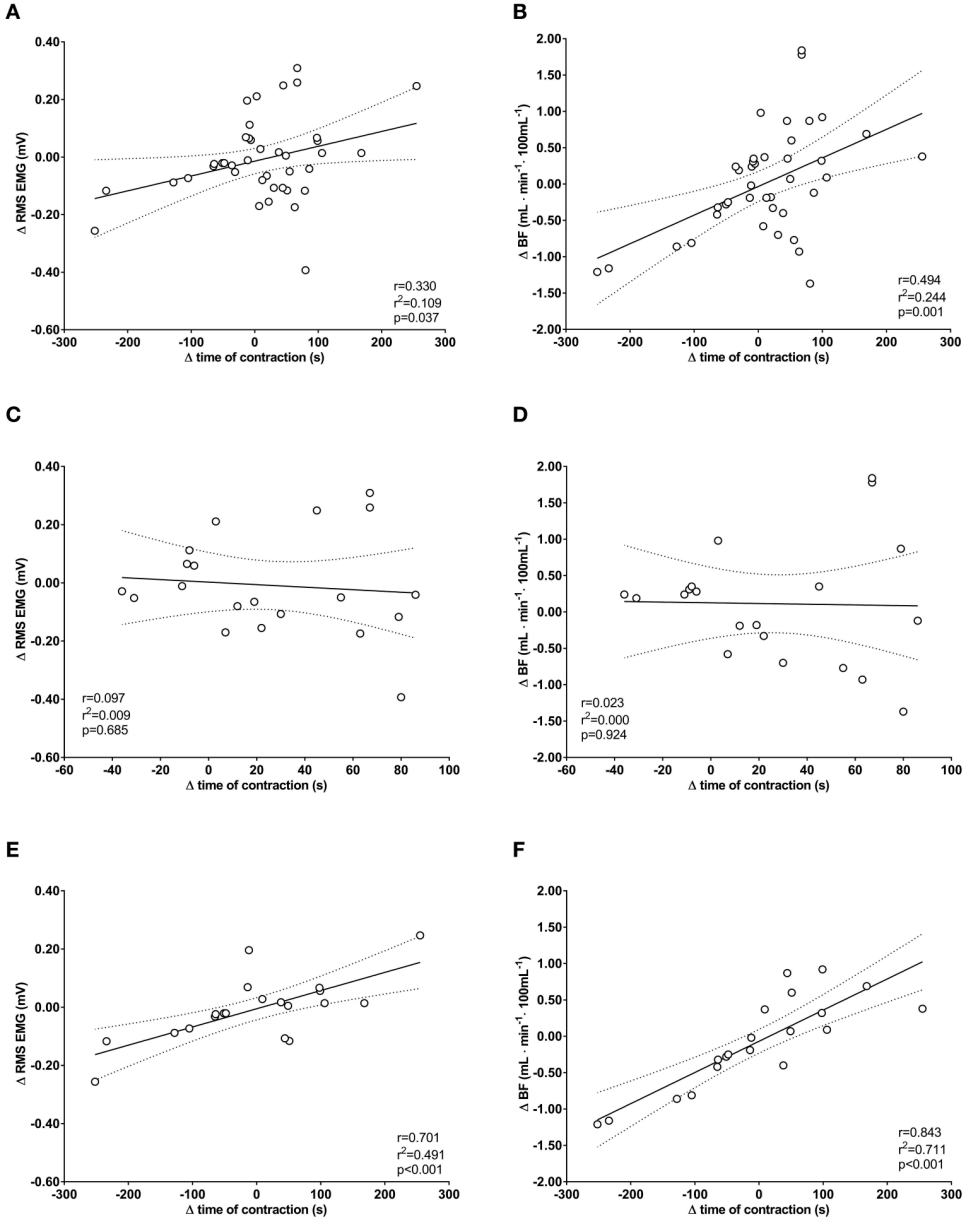
Univariate correlations between changes (Δ) in time of sustained isometric contraction at 30% MVIC torque and changes in EMG amplitude (Δ RMS EMG) and perfusion (Δ BF_m_) of quadriceps femoris muscle in the postoperative period. Plots are for pooled group data **(A,B)** and separately for BFR group **(C,D)** and SHAM-BFR group **(E,F)**.

The univariate correlation of the pooled group data between of Δ time of contraction and Δ RMS EMG was low (*r* = −0.330; *r*^2^ = 0.109) and significant (*p* = 0.038). The correlation with Δ BF_m_ was moderate (*r* = 0.494; *r*^2^ = 0.244) and significant (*p* = 0.001) (Figures 3A,B). For BFR group, the correlation between Δ time of contraction and Δ RMS EMG was low (*r* = −0.099; *r*^2^ = 0.010) and non-significant (*p* = 0.679); also, the correlation with Δ BF_m_ was low (*r* = −0.023; *r*^2^ = 0.001) and non-significant (*p* = 0.922) (Figures 3C,D). For SHAM-BFR group, the correlation between Δ time of contraction and Δ RMS EMG was high (*r* = 0.701; *r*^2^ = 0.491) and significant (*p* < 0.001); likewise, the correlation with Δ BF_m_ was high (*r* = 0.843; *r*^2^ = 0.711) and significant (*p* < 0.001) (Figures 3E,F).

## Discussion

This is the first evidence of preventative effect of low-load BFR exercise on skeletal muscle endurance following ACL reconstruction. The results showed that the patients treated with low-load BFR exercise protocol did not display deterioration in QF muscle endurance in the first 4 weeks after surgery, whereas patients who had performed the same exercise protocol with sham occlusion demonstrated ~50% reduction of muscle endurance. The difference in QF endurance between groups was no longer evident 12 weeks after surgery, thus the main hypothesis is only partially confirmed. As shown by multivariate regression models of changes in time of sustained isometric contraction, the decrease in QF muscle endurance was moderately correlated with decreases in BF_m_ (Figures 3A,B); the latter was also the most influential and significant individual predictor of change in QF endurance during the 12-week postoperative period. The preconditioning had neither significant nor a clinically important positive effect on QF maximal strength at any time point after surgery, which corroborates the results of our first study with a similar protocol of intervention (Grapar Zargi et al., 2016). From the parameters measured in the present study, we cannot identify explicit physiological mechanisms for these findings, however some plausible explanations can be given.

### Muscle blood flow and microvascular function

A ~50% increase in post-exercise muscle blood flow in BFR group and a ~30% decrease in the SHAM-BFR group at 4 weeks after the reconstruction allude to changes in either vasomotor tone regulation or density of muscle capillary network. It appears that preconditioning with BFR exercise enhanced muscle microvascular function and preserved it up to 4 weeks after surgery, whereas deterioration of microvascular function was present in patients who were preconditioned with sham intervention. Degradation of skeletal muscle capillary network follows prolonged ischemia (Hammersen et al., 1979) and delays its recovery (Appell et al., 1993), therefore deterioration of muscle blood-flow response to exercise observed in the SHAM-BFR group could be a sign of the ischemia-reperfusion damage to the QF muscle microcirculation induced by the surgical tourniquet. The changes in [tHb] acquired by near-infrared spectroscopy, on which the measurement of BF_m_ in our study is based on, have been previously shown to be negatively correlated (*r*^2^ = 0.58, *p* = 0.003) with muscle protein oxidation after release of surgical tourniquet and can be thus regarded as a valid predictor of ischemia-reperfusion muscle injury (Shadgan et al., 2012).

To our knowledge, no other reports of the long-term effects of preconditioning with low-load BFR exercise on atrophied muscle microvascular function or hemodynamics exists in the literature. In healthy subjects, Patterson and Ferguson (2010) reported a similar augmentation (38–47%) of muscle blood flow following a 4-week low-load BFR training of plantar flexors, despite utilizing a different method of blood flow assessment (i.e., post-occlusive hyperemia). The present results are also in line with our previous observations of improved QF muscle oxygenation following a 4-week low-load BFR exercise program, where a 120% increase in Δ[tHb], an index of gross muscle perfusion, and concomitant 56% increase in Δ[O_2_Hb] was noted (Kacin and Strazar, 2011). More than 2-fold higher magnitude of increase can be attributed to a much longer training period and the fact that blood flow was reevaluated immediately after the intervention. Interestingly, very similar effects on muscle blood flow have been also reported with the use of standard IPC in healthy subjects. Different doses of IPC, ranging from very frequent application of 6 cycles of 5-min arterial occlusion and 5-min reperfusion daily for 4 weeks (Kimura et al., 2007) to less frequent application of 4 cycles of 5-min arterial occlusion and 5-min reperfusion 3 times weekly for 8 weeks (Jones et al., 2015) have been proven effective for improving forearm blood flow and skin microcirculatory function. Shorter interventions of 4 cycles of 5-min arterial occlusion and 5-min reperfusion daily for 7 days, have also been shown to have a similar protective effect (Jones et al., 2014). A recent study by Jeffries et al. (2018) demonstrated that the same 7-day IPC protocol increases oxidative function of plantar flexor muscles by ~13% and induces faster muscle reoxygenation (~32–43% increase in time constant of [O_2_Hb]) following repeated isometric exercise of comparable submaximal intensity (40–60% MVIC). The latter were attributed to either an enhanced oxidative capacity or increased oxygen delivery via a vasodilatory mechanism or vascular adaptation (Jeffries et al., 2018). However, the exact mechanisms of enhanced microcirculatory function of skeletal muscle by IPC have not been fully identified as yet. It was shown on a rat model of mesentery microcirculation that reduction of leukocyte-endothelial interaction in postcapillary venules plays an important role in this regard (Erling et al., 2010). In humans, repetition of IPC stimulus was also shown to augment myocardial blood flow via endothelium-dependent vasodilation through increases in nitric oxide production (Kimura et al., 2007).

The similarity in effects on muscle endurance and microvascular function observed by standard IPC and our present results confirms the initial presumption that the IPC protocol and low-load BFR exercise impose a comparable stimulus to the skeletal muscle, at least regarding muscle microvascular function and adaptation. However, not every protocol of BFR exercise may be equally effective. Proper combination of cuff width and pressure, occlusion time, duration of reperfusion between sets and the timing of its application is likely the key to eliciting protective effects by BFR exercise. The protocol utilized in the present study comprised 6 sets of low-load exercise under occlusion at 150 mmHg with 90 s reperfusion in-between, which gives a total of 3 cycles of ~180 s under occlusion delivered 5 times in 8 days, indicating that a significant effect on muscle microcirculatory function was achieved by substantially shorter overall ischemia-reperfusion exposure than during 7-day IPC protocols used by other researchers (Jones et al., 2014; Jeffries et al., 2018). The exact reason for the enhanced efficiency cannot be elucidated, but we presume that much higher metabolic rate and metabolite accumulation in the muscle due to its activity potentiate the ischemic stimulus.

By performing the last BFR exercise session within 24–48 h prior to surgery, we utilized the same time frame as for the late phase of protection with IPC (Loukogeorgakis et al., 2005). The attenuated decrease in BF_m_ strongly suggest that QF muscle microcirculatory function was successfully protected in patients preconditioned with BFR exercise. This presumption is further corroborated with the results of the multivariate regression model showing that deterioration of QF muscle endurance in SHAM-BFR group was strongly correlated with the reduction in BF_m_ (Figures 3E,F), whereas no significant correlation was found in the BFR group (Figures 3C,D). The reason for the difference in pattern of association of variables between the groups is unknown. It is possible that a large difference in magnitude of change in dependent variable between the groups has influenced the sensitivity of the model. It must also be considered that the general robustness of the model may not have been sufficient for detecting actual association between the variables.

### Muscle activation

No reports on long-term effects of BFR exercise on median frequency in either healthy or injured subjects can be found in the literature. There are, however, several reports on acute frequency modulation induced by different protocols of BFR that may be relevant for interpretation of our results. Previous studies, investigating the interaction between BFR exercise and patterns of neuromuscular activation, have shown that acute BFR exercise augments neural activation and recruitment of type II motor units (Moritani et al., 1992; Takarada et al., 2000; Moore et al., 2004). Pierce et al. (2006), and more recently also Fatela et al. (2016), demonstrated that higher levels of BFR elicited greater decrements in median frequency of rectus femoris, vastus lateralis, and vastus medialis muscle in a short period after exercise, which implies that the magnitude of neuromuscular fatigue is substantially larger following this form of exercise. A progressive increase in type II motor unit recruitment and reduction in fatigue would therefore be expected to be the primary long-term adaptation of BFR training. However, the uniform decrease in F_med_ of vastus medialis muscle during isometric endurance test does not suggest any differences in recruitment of type I and II motor units, or reduction in neuromuscular fatigue, between our patient groups at any time point after surgery. It must be emphasized that the F_med_ was decreased although the same absolute torque was sustained during the test. Given that QF muscle MVIC torque was reduced by ~20% in both groups at both POST WK4 and WK12, the relative intensity of muscle contraction during the postoperative endurance tests was correspondingly increased. Median frequency normally increases proportionally to the increase in relative intensity of muscle contraction (Croce et al., 2014), but the opposite was observed in our patient cohort. Similar paradox of decreased median frequency of the QF muscle in patients following ACL reconstruction was also observed in other studies (McHugh et al., 2001; Drechsler et al., 2006), which implies a pathological pattern of motor unit recruitment following surgery. The altered neuromuscular activation is most likely driven by altered sensory input from the damaged joint and soft tissue, which contributes to the phenomenon of arthrogenic muscle inhibition (Hurley et al., 1994).

Despite the uniform decrease in F_med_ after the surgery, BFR group presented significant increase in the amplitude of RMS EMG at POST WK4. This implies on the preserved ability to increase volitional activation of larger number of motor units to compensate for the decrease in force capacity, which in turn resulted in significantly longer times of sustained contraction compared to SHAM-BFR group. The results are in line with consensus in the literature that BFR exercise implicates greater muscular activation to maintain the same work output (Moritani et al., 1992; Takarada et al., 2000; Wernbom et al., 2009; Yasuda et al., 2009; Cook et al., 2013; Fahs et al., 2015; Loenneke et al., 2015; Fatela et al., 2016). Hypothetically, these increments in EMG amplitude may reflect an enhanced recruitment of type II motor units (Moritani et al., 1992; Takarada et al., 2000; Moore et al., 2004) due to the local accumulation of metabolites (Loenneke et al., 2015) or increase in eccentric muscle activity (Wernbom et al., 2009) during exercise with BFR. In face of no difference in F_med_ between the groups, an increased portion of active type II motor units in BFR group following surgery cannot be clearly confirmed or refuted. This paradox needs to be scrutinized by future experiments.

### Potential changes in central motor drive

The increase in QF muscle endurance and RMS EMG amplitude observed in the BFR group may be also explained by modulations in central motor drive. Given that neither subjective evaluation of perceived body exertion nor any direct measure of central activation ratio were used in the present study, the potential mechanisms discussed below must be regarded as speculative.

Similar 3–8% improvements in endurance performance have been reported immediately after application of standard IPC protocol in cats (Phillips et al., 1997) and healthy humans (Chrisafulli et al., 2011; Cruz et al., 2015; Patterson et al., 2015; Griffin et al., 2018). Several authors hypothesized that increased muscle endurance observed after IPC may be attributed to enhanced ability to utilize central reserve, which represents unused potential of muscle activation from higher motor centers (Chrisafulli et al., 2011; Cruz et al., 2015; Patterson et al., 2017). The IPC supposedly decreases sensitivity of type III and IV polymodal receptors in the muscle, which enables additional activation of central reserve and subsequently preserves muscle force production (Cruz et al., 2017). Given that BFR exercise, especially if performed to volitional failure, induces greater volitional effort and muscle discomfort than a free-flow exercise, it may enhance utilization of central reserve and decrease central fatigue by similar mechanisms as hypothesized for IPC. Given that the central fatigue has been accounted for up to 65% of the decrease in force production during sustained muscle contraction of similar intensity as used in our endurance test (Smith et al., 2007), a decrease in central fatigue may have played an important role in enhanced isometric QF endurance of the patients preconditioned with BFR exercise.

### Limitations and prospects of future research

The following limitations need to be considered when interpreting the reported findings. Firstly, there are several equally important initial patients' characteristics (level of physical activity, leg-dominance, preoperative muscle deficits, genetic muscle fiber type ratio, pain intensity and apprehension level, etc.) that need to be considered when randomizing patients into groups. Combining groups by all potential confounding factors is virtually impossible in a clinical setting, thus differences between groups in some of the characteristics may have interfered with our results. Secondly, the extent of ischemic-reperfusion injury of QF muscle cannot be precisely quantified in the absence of humoral or tissue markers. It must be emphasized, however, that accurate estimation of small-scale ischemic-reperfusion injury of the QF muscle after ACL reconstruction is methodologically very demanding as inflammatory factors and markers of muscle cell injury released by ischemic-reperfusion injury are heavily masked by concomitant release of high concentrations of overlapping markers of tissue damage elicited by surgery *per se*. Thirdly, the increased muscle blood flow in the operated leg could be also influenced by postoperative inflammation of the knee joint and surrounding soft tissues. This seems, however, very unlikely, as no swelling, redness or increased temperature was noted in any of our patients at the site of measurement; the NIRS optode was placed at least 15 cm proximal to the affected joint, which is outside the wider inflammation area. Fourthly, the assessment of training gains in muscle function immediately after the BFR exercise intervention is lacking. To maximize the protective effect of IPC, last exercise unit had to be scheduled 24–48 h before the surgery, which made the in-between testing neither scientifically justified nor logistically feasible. The immediate effects of the preconditioning intervention thus remain unknown. Lastly, the regression models reported in the present study lack robustness as they are built on rather small number of observations.

Evaluation of molecular mechanisms of vasodilatory or vascular adaptation in the muscle following BFR exercise need to be further investigated. For more certain exclusion of the influences of inflammation on muscle blood flow, especially in the early postoperative period, capillary network density and presence of tissue inflammatory factors should be evaluated from muscle biopsies. Both peripheral and central neural mechanisms of enhanced muscle activation must be scrutinized in depth, with emphasis on modulation of pain and exertion thresholds during exercise. Future research should focus also on optimizing the protocol of preconditioning with low-load BFR exercise and timing of its application before surgery.

### Physiological relevance and clinical implications

This is the first study to demonstrate that short-term preconditioning with low-load BFR exercise training has significantly stronger positive effect on QF muscle endurance, its activation and perfusion after ACL reconstruction than equal training performed without blood flow restriction. However, both training regimes failed to affect the deterioration of maximal QF muscle strength following surgery. The preserved endurance of QF muscle was paralleled with enhanced muscle perfusion and increased amplitude of EMG for a given torque output. The latter corroborates the paradigm of enhanced volitional drive to the working muscles after IPC observed in athletes (Cruz et al., 2017). Given that some individuals with reconstructed ACL can have chronic 10% deficit in central activation ratio and 30% lower maximal EMG amplitude of QF muscle (Pamukoff et al., 2017), even small improvements in central motor drive induced by low-load BFR exercise would greatly increase QF muscle performance in these patients.

The importance of muscle perfusion in maintaining local muscle endurance after ACL reconstruction is a novel finding, which is in line with similar effects on muscle oxygenation and hemodynamics seen after low-load BFR training (Patterson and Ferguson, 2010; Kacin and Strazar, 2011) and IPC application (Jones et al., 2014, 2015; Jeffries et al., 2018) in healthy subjects. Based on the present results we presume that enhanced muscle perfusion after BFR training was a result of attenuated degradation of capillary network and preserved microcirculatory function in the atrophied muscle. Given that pre-operative muscle endurance has been shown to be the strongest independent predictor of QF muscle weakness following ACL reconstruction (Grapar Žargi et al., 2017), the preconditioning with low-load BFR exercise may serve as a valuable addition to standard physiotherapy programs for patients elected for ACL reconstruction, or other limb surgery performed with a tourniquet.

## Author contributions

Conceived and designed the research, Interpreted the experimental results, and Approved the final version of the manuscript: TGŽ, MD, KS, and AK; Performed the experiments, Analyzed the data, and Drafted the manuscript: TGŽ and AK; Prepared the figures: AK; Edited and revised the manuscript: TGŽ, MD, and AK.

### Conflict of interest statement

The authors declare that the research was conducted in the absence of any commercial or financial relationships that could be construed as a potential conflict of interest. The reviewer OJ and handling Editor declared their shared affiliation.
